# Relationship between the Asthma Control Test (ACT) and other outcomes: a targeted literature review

**DOI:** 10.1186/s12890-020-1090-5

**Published:** 2020-04-03

**Authors:** Bas C. P. van Dijk, Henrik Svedsater, Andreas Heddini, Linda Nelsen, Janita S. Balradj, Cathelijne Alleman

**Affiliations:** 1 Pharmerit International, Rotterdam, The Netherlands; 2 Value Evidence and Outcomes, GlaxoSmithKline plc., Brentford, UK; 3 Medical Affairs, GlaxoSmithKline Nordic Region, Stockholm, Sweden; 4 Value Evidence and Outcomes, GlaxoSmithKline plc., Collegeville, PA USA

**Keywords:** Asthma Control Test, ACT, Lung function, Quality of life, Rescue medication, Exacerbations

## Abstract

**Background:**

The Asthma Control Test (ACT) has been used to assess asthma control in both clinical trials and clinical practice. However, the relationships between ACT score and other measures of asthma impact are not fully understood. Here, we evaluate how ACT scores relate to other clinical, patient-reported, or economic asthma outcomes.

**Methods:**

A targeted literature search of online databases and conference abstracts was performed. Data were extracted from articles reporting ACT score alongside one or more of: Asthma Control Questionnaire (ACQ) score; rescue medication use; exacerbations; lung function; health−/asthma-related quality of life (QoL); sleep quality; work and productivity; and healthcare resource use (HRU) and costs.

**Results:**

A total of 1653 publications were identified, 74 of which were included in the final analysis. Of these, 69 studies found that improvement in ACT score was related to improvement in outcome(s), either as correlation or by association. The level of evidence for each relationship differed widely between outcomes: substantial evidence was identified for relationships between ACT score and ACQ score, lung function, and asthma-related QoL; moderate evidence was obtained for relationships between ACT score and rescue medication use, exacerbations, sleep quality, and work and productivity; limited evidence was identified for relationships between ACT score and general health-related QoL, HRU, and healthcare costs.

**Conclusions:**

Findings of this review suggest that the ACT is an appropriate measure for overall asthma impact and support its use in clinical trial settings.

GlaxoSmithKline plc. study number HO-17-18170.

## Background

Asthma is a common and treatable disease that can impact heavily on health-related quality of life (HRQoL) [1]. Medical experts agree that the level of asthma control is a key feature when determining the best asthma treatment required [1, 2]. Developed by asthma experts, the Asthma Control Test (ACT) provides a numerical score to assess the control of asthma [3]. It comprises five questions regarding aspects of asthma control relevant to patients. The ACT assesses frequency of shortness of breath, night-time/early awakenings, rescue medication use, overall asthma control, and loss of productivity. Each question is answered on a 5-point scale, with a total score ranging from 5 to 25; higher scores indicate improved asthma control [2, 3]. A score of ≥ 20 indicates “well-controlled” asthma, while a score < 20 indicates asthma that is “not well controlled”. The ACT provides patients with asthma and their doctors and nurses with a useful measure to help determine the level of treatment required [2, 3]. It has been tested extensively in patients with asthma [4], clinically validated against spirometry and specialist assessment [3], and is recognized by the National Institutes of Health since its 2007 asthma guidelines [2]. Despite its clinical utility, a need remains to assess the link between ACT score and asthma treatment benefits and outcomes, and its suitability as an endpoint in clinical trials. Previous studies have used the ACT as a measure of response to treatment [5, 6], including a recent Phase III study that was not published in time to be included in this review [7]. The aim of the current study was to assess the extent to which ACT score is correlated, or associated, with other important clinical, patient-reported, and economic asthma outcomes.

## Methods

**Fig. 1:**
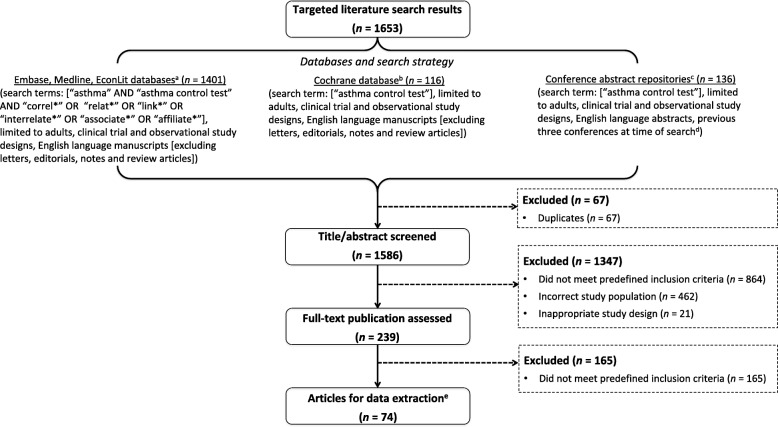
Flow diagram of search strategy and targeted literature review approach. ^a^Articles published before February 9th, 2017 were captured in the EMBASE, MEDLINE and EconLit database searches. ^b^The Cochrane database search reviewed articles published prior to January 21st, 2017. ^c^Conference abstract repositories searched were the American Thoracic Society (ATS), European Respiratory Society (ERS) and American College of Chest Physicians (ACCP) CHEST. ^d^Conference databases were searched for the last three editions of the conference in question (i.e. 2015–2017 or 2014–2016), up to January 21st, 2017. ^e^Studies could have reported on multiple outcomes. *ACT*: Asthma Control Test; *HRQoL*: health-related quality of life; *QoL*: quality of life

A targeted literature search of the EMBASE, MEDLINE, EconLit, and Cochrane databases was performed, in addition to searching the relevant conference abstract repositories of the American Thoracic Society (ATS), European Respiratory Society (ERS), and American College of Chest Physicians (CHEST). Articles published before February 9th, 2017 were captured in the EMBASE, MEDLINE and EconLit database searches, while searches of the Cochrane database and conference repositories reviewed articles and congress abstracts published prior to January 21st, 2017. The details of the search strategy are included in Fig. 1.

Identified publications were initially screened for eligibility by title and abstract, and full-text articles of all eligible studies were then assessed. Eligible studies included human studies investigating adult patients (≥ 18 years old) with a primary asthma diagnosis. Articles reporting the results of observational studies, clinical trials, longitudinal/cross-sectional studies and other studies reporting relationships between ACT score and other outcomes of interest were included, while letters, editorials, notes, and review articles were excluded.

Articles that met predefined inclusion criteria were retained for full text review i.e. articles were included if they reported the results of studies investigating a relationship between ACT score and/or asthma severity and one or more of identified outcomes of interest: symptom control, use of rescue medication, exacerbations, pulmonary function, HRQoL/utilities, sleep quality, productivity and activity levels, and resource use and costs.

Following identification of articles eligible for full-text review, the number of articles assessing each relationship, as well as the strength, significance, and direction of those relationships, was quantified. We also evaluated the extent to which the tested relationships differed per ACT score and/or asthma severity.

Articles describing a statistical relationship between two continuous variables were defined as reporting a “correlation”; statistical tests used in these studies included Pearson’s chi-square test and the Spearman correlation test. Those that instead reported a trend between subgroups or over time, assessed by covariance tests, regression analysis, or empirical observation, were defined as reporting “associations”; in other words, these are assessments of the extent to which two measures co-vary in an expected manner (i.e. an improvement of one measure should represent an improvement in another outcome measure).

Data for each outcome of interest were extracted from the targeted studies by means of a formalized extraction grid. Bibliographic and methodologic details of the study, basic population characteristics, and ACT scores at baseline and later if applicable were extracted. For each outcome of interest that was examined for a relationship with ACT score, the following were included: a description of the outcome and its value; time of measurement (baseline/later); change from baseline; method used to test relationship and the outcome of the test; significance level (range, 95% confidence interval, or *p*-value); statistical significance of relationship (yes/no); and the direction of the relationship (positive/negative).

## Data availability

All publications identified in the targeted literature review were available from public databases (EMBASE, MEDLINE, EconLit, and the Cochrane Library), and can be accessed there or on the articles’ respective journal websites. Conference abstracts were available from the abstract repositories of the professional organizations that arranged the conferences in question (the ATS, ERS, and CHEST). Relevant data were extracted from these publications, but no new data were generated within the course of this literature review. Accordingly, no databases or data repositories were created. The protocol for this literature review is not publicly available.

## Results

The targeted literature search identified 1653 publications (1401 in EMBASE, MEDLINE, and EconLit; 116 in Cochrane databases; and 136 in conference abstracts), of which 67 were duplicates. Of the 1586 unduplicated publications screened, 239 were reviewed in full and 74 were included in the final analysis (Fig. 1). The analysis found that in 68 publications an improvement in ACT score was correlated or associated with improvements in key outcomes of interest (Table 1). Studies assessing symptom control, healthcare resource use, or lung function were among the most commonly identified; fewer studies assessing QoL, sleep, and productivity were found. Asthma severity was not frequently reported.

**Table 1:** Correlation of ACT-measured improvement with improvement in other outcomes

Outcome	Number of studies, *n*^a^	Studies reporting correlation, *n* (*n* reporting statistical significance)	Studies reporting association, *n* (*n* reporting statistical significance)
Asthma Control Questionnaire	7	6^8–13^ (5)^8–12^	1^14^ (0)
Use of rescue medication	10	2^15,16^ (2)^15,16^	8^17–24^ (4)^17,18,22,23^
Number of asthma exacerbations	10	2^26,27^(2) ^26,27^	8^17,22,23,28–32^ (7)^22,23,28–32^
Lung function	17	8^3,15,27,33–37^ (5^b^)^3,34–37^	9^11,13,18,38–43^ (7^b^)^11,13,38–40,42,43^
General HRQoL	7	0 (0)	7^49–55^ (5^b^)^49,50,52–54^
Asthma-related QoL	7	5^28,34,44–46^(5)^28,34,44–46^	2^47,48^ (2)^47,48^
Sleep quality	6	2^45,56^(2)^45,56^	5^56–60^ (2^b^)^58,59^
Work and productivity	7	1^63^ (1)^63^	6^49,51,53,55,64,65^ (2)^49,65^
Healthcare resource use	17	1^63^ (0)	16^47,49,51,53–55,67–76^ (8^b^)^54,55,67,69,71,72,74,75^
Healthcare costs	6	0 (0)	6^50,65,70,76–78^ (1^b^)^77^

“Correlation” denotes a direct, statistically tested relationship between ACT score and the outcome. Statistical analyses included the Pearson’s chi-square test and the Spearman correlation test. “Associations” are defined as trends between subgroups or over time, assessed by covariance tests, regression analyses, or empirical observation. *ACT* Asthma Control Test, *HRQoL* health-related quality of life, *QoL* quality of life

^a^Number of studies reporting on a particular outcome is presented per row; studies could report multiple outcomes within one topic

^b^Publications that did not assess the significance were not taken into account for this calculation

### ACT score and asthma symptom control

In total, seven publications reported ACT and Asthma Control Questionnaire (ACQ) data (Table 1) [8–14]. Six publications found strong and consistent correlations between improvement in ACT score and improvement in ACQ score [8–13], with five of the publications assessing the statistical significance of the correlations. All five of these reports found statistically significant relationships between ACT and ACQ scores (*p* < 0.05) [8–12]. Schuler et al. [12] reported that the ACQ-7 had a strong positive correlation, with a moderate correspondence with ACT score (Cohen’s kappa κ = 0.56). Zhou et al. [13] reported strong correlations between improvement in ACT score and improvement in both the ACQ-7 (all items) (Spearman correlation coefficient, *r* = − 0.687; robust correlation, *r* = − 0.865) and the ACQ-6 (excluding spirometry; Spearman correlation coefficient, *r* = − 0.491; robust correlation, *r* = − 0.637), although statistical significance was not tested. A further publication reported a similarly moderate effect with the same measure of concordance (κ = 0.52) as Schuler et al., without testing for significance [14].

### ACT score and rescue medication use

Almost all of the 10 publications reporting on ACT score and rescue medication use [15–24] found a relationship between worsening ACT scores and increasing short-acting beta agonist (SABA) or rescue inhaler use (Table 1). Weak but statistically significant correlations between ACT score and the number of SABA inhalers dispensed were identified in two publications (*ρ* = − 0.33, *p* = 0.001 in both); these studies may have been reporting on the same population [15, 16]. One study reported strong relationships between risk of excess SABA use and both ACT score and change in ACT score; a 2-point worsening in ACT score led to a 46% increased risk of excess SABA use [22]. Another publication showed that the odds of having ≥6 SABA inhaler dispensings increased markedly at lower ACT scores over a continuous range of ACT scores [23].

Three publications reported SABA use by asthma control subgroup, and demonstrated trends between lower ACT score and higher rescue inhaler use [18, 20, 24], with one study performing a regression analysis (*r* = 0.51, *p* < 0.001) [18].

Only one study was identified that evaluated the relationship between ACT score and rescue medication-free days. Improvement was evaluated by both a change from baseline and the proportion of patients who achieved asthma control, similar to the Salford Lung Study trial design [25]. The results suggested that improvement in ACT score was related to improvement in rescue medication-free days [19].

### ACT score and asthma exacerbations

Ten studies reported on the relationship between improvement in ACT score and reduction in asthma exacerbations (Table 1) [17, 22, 23, 26–32].

Across the studies, there was some variation in the definitions used to characterize an exacerbation. However, these definitions were generally comparable, specifying an exacerbation as a worsening in asthma symptoms that required one or more of oral/systemic corticosteroid use, hospitalization, or an emergency healthcare visit.

Two of the studies tested for correlations between improvement in ACT score and reduction in asthma exacerbations, resulting in small but significant correlation coefficients of − 0.129 and − 0.349, respectively (*p* < 0.01) [26, 27].

In the other eight publications, fewer exacerbations were observed in patients with higher ACT scores [17, 22, 23, 28–32], with seven of these publications reporting a statistically significant relationship between higher ACT score and lower numbers of exacerbations in patients split into the various ACT subgroups [22, 23, 28–32].

### ACT score and lung function

Twenty-five articles were identified which assessed the relationship between ACT score and lung function. Of these, eight publications did not fully meet the predefined inclusion criteria and were therefore subsequently excluded from data extraction.

There is a strong body of evidence supporting a relationship between improvement in ACT score and improvement in lung function, particularly with respect to forced expiratory volume in 1 s (FEV_1_; Table 1). In total, 17 publications detailing studies that met the inclusion criteria reported lung function measurements [3, 11, 13, 15, 18, 27, 33–43], with some reporting multiple outcomes (e.g. FEV_1_ and forced vital capacity [FVC]) [15, 27, 36, 38].

Of the 14 publications reporting FEV_1_, seven reported statistically tested correlations between improvement in ACT score and improvement in FEV_1_ [3, 15, 27, 34–37]. Of these, five demonstrated statistically significant correlations, with coefficients ranging from 0.177 to 0.518, as calculated by different methods [3, 34–37]. This evidence was supported by the remaining seven studies, which tested the relationships between ACT score and FEV_1_ by linear regression, analysis of variance (ANOVA), or in subgroups of patients categorized according to ACT score or FEV_1_ [11, 13, 18, 38–40, 43]; of these seven articles, six reported statistically significant relationships [11, 13, 38–40, 43].

Three articles tested for a statistical correlation between improved asthma control as measured by increased ACT score and improvement in FVC [15, 33, 36], with only one demonstrating a notable relationship (correlation coefficient, *ρ* = 0.26, *p* = 0.01) [36]. The one publication that assessed FVC without a correlation test also reported a strong relationship (*p* = 0.000) [42].

### ACT score and QoL

A total of seven articles reported HRQoL in asthma patients [28, 34, 44–48], all of which showed strong positive relationships between improvement in ACT score and improvement in Asthma Quality of Life Questionnaire (AQLQ) score (Table 1). All seven studies published statistically significant results, five from correlation tests [28, 34, 44–46], and two derived from regression analyses [47, 48].

In total, seven studies reported general HRQoL in ACT subgroups, measuring HRQoL by the Short Form (12-item) Health Survey (SF-12) or the EuroQol five dimensions questionnaire (EQ-5D) [49–55]. In both questionnaires, a higher score indicates improved QoL.

For SF-12, a consistent trend was observed that individual physical and/or mental component scores were lower for the groups with asthma that was “not well controlled”. Statistically significant differences in individual SF-12 domain scores between patients with uncontrolled/not well-controlled versus controlled asthma (ACT score thresholds varied) were observed in three (physical component) [49, 53, 54] and two (mental component) studies [49, 52], respectively.

Guilbert et al. [54] performed bivariate and multivariate analyses on the relationship between asthma control (“well-controlled” [ACT score > 19] vs. “not well-controlled” [ACT score ≤ 19]) and SF-12 physical domain score, observing a negative relationship between ACT score ≤ 19 and SF-12 score. The mean differences in SF-12 scores between patients with “not well-controlled” and “well-controlled” asthma were − 7.0 and − 3.4 for the bivariate and multivariate analyses, respectively (both *p* < 0.001) [54].

Additionally, one study reported a significant difference in EQ-5D between patients with “not well-controlled” (ACT score < 20) and “well-controlled” (ACT score ≥ 20) asthma (EQ-5D scores, 0.7 vs. 0.9; *p* < 0.0001) [50]. This finding is of particular interest, given that the EQ-5D system may lack sensitivity which often does not correlate with underlying clinical measures [56, 57].

### ACT score and sleep quality

In total, six articles reported on aspects of sleep quality, including instruments that measured daytime sleepiness and obstructive sleep apnea [45, 58–62]; three of these studies utilized multiple sleep quality instruments (Table 1) [58, 60, 62].

Of the four studies using the Pittsburgh Sleep Quality Index (PSQI) [45, 58, 60, 62], two reported notable, statistically significant correlations between improvement in ACT score and improvement in sleep quality (r = − 0.315, *p* < 0.001 and r = − 0.620, *p* < 0.001, respectively) [45, 58]. Additionally, Lv et al. [60] found that poor sleep quality, assessed by PSQI, was related to lower ACT score using regression analyses (β = − 0.87, *p* = 0.045).

A statistically significant relationship was also demonstrated between improvement in ACT score and improvement in sleep quality measured using the Medical Outcomes Study (MOS) Sleep Scale. Compared with patients who had an ACT score ≥ 20, those with a score < 20 had significantly higher scores in all components of the MOS Sleep Scale (all *p* < 0.001, F-values 19.1–109.0), corresponding to poorer sleep quality [61].

A relationship between the level of asthma control, as measured by ACT score, and improvement in sleep quality, as measured by the Sleep-5 questionnaire, was also reported, but the relationship was not tested for statistical significance [59].

Two studies reported that patients with lower ACT scores tended to have higher (worse) Epworth Sleepiness Scale scores, although no statistical analysis was performed [58, 62].

Regression analyses (unadjusted and adjusted) were performed in one study to determine the relationship between asthma control (measured by the ACT) and the Berlin Questionnaire for obstructive sleep apnea [60].

### ACT score and productivity and activity levels

Out of a total of seven articles, five reported on the relationship between improvement in ACT score and improvement in productivity, as measured by the Work Productivity and Activity Impairment questionnaire (WPAI) (Table 1) [49, 51, 53, 55, 63]. One study reported a negative correlation between ACT score and WPAI score (correlation varied between − 0.707 and − 0.750, statistical significance not reported) [63]. The other four studies reported higher work impairment in patients whose asthma was not well controlled (ACT score < 20), compared with those with well-controlled asthma (ACT score ≥ 20) [49, 51, 53, 55]. However, only one of these found that relationship to be statistically significant [49].

Two studies reported on the relationship between the level of asthma control, as measured by ACT score, and improvement in productivity using other measures (i.e. the Effort-Reward-Imbalance questionnaire, the Sheehan Disability Scale, and the Impact on Work Productivity Index [IMPALA]) [56, 57]. Of these, one study reported a statistically significant relationship between the subgroup with improved asthma control and productivity measured by the Sheehan Disability Scale and IMPALA [57].

### ACT score and resource use and costs

In total, 17 publications reported on the relationship between ACT score and HRU (Table 1) [47, 49, 51, 53–55, 64–74].

One study reported a non-significant relationship between improvement in ACT score and both the ratio of maintenance to reliever medication dispensed, and inhaler nebulization rates [66].

A total of 16 studies examined relationships between reduction in unscheduled care and ACT score improvement from “not well controlled” to “well-controlled” asthma [47, 49, 51, 53–55, 67–76]. Statistically significant results were reported for unscheduled outpatient clinic visits by two studies [54, 74], for emergency department visits by five studies [54, 55, 71, 74, 75], for hospitalizations by three studies [55, 71, 72], for ‘urgent health care utilization’ by one study [69] and use of inhaled corticosteroids by one study [67].

In total, six studies reported on costs [50, 65, 70, 76–78], of which five reported relationships between asthma control subgroup and direct medical costs [50, 65, 70, 76, 77], and three with indirect medical costs [65, 77, 78]. However, only one study reported statistically significant results for direct medical costs [77].

The average cost (Euros [€)/month/patient) of well-controlled asthma versus “not well-controlled” asthma was reported as €28 versus €140 in France and €77 versus €252 in Spain [50]. In Spain, indirect costs were significantly higher in older patients (41–65 years, €405.08), patients with more severe disease (€698.95), and patients with more poorly controlled asthma (€466.86) [65]. Mean per-patient annual costs of asthma management for patients with derived ACT scores of < 15, 15–19 and ≥ 20 were reported as, in the Asia-Pacific, US$861, US$319 and US$193 [70]; and in Europe, €1604, €512, and €232 [76]. Patients with asthma control spent S$48 (US $36) more per doctor visit on asthma drugs (*p* < 0.01) but incurred S$65 (US$48) less per doctor visit in total costs (*p* < 0.01) than those with suboptimal asthma control [77].

The data suggest that asthma that was not well controlled (ACT score < 20) led to higher direct medical costs [65, 77], higher unscheduled care costs [70, 76], and higher total societal costs of asthma [50], while data on indirect costs suggest that “not well-controlled” asthma (ACT score < 20) leads to higher indirect medical costs [77, 78], and higher cost of workdays lost [65].

## Discussion

This review aimed to qualitatively assess the link between ACT score and key asthma outcomes through a targeted review of the available literature. Substantial evidence was identified for relationships between ACT score and ACQ score, lung function, and asthma-related QoL; moderate evidence was obtained for relationships between ACT score and rescue medication use, exacerbations, sleep quality, and work and productivity; limited evidence was identified for relationships between ACT score and general health-related QoL, HRU, and healthcare costs. While links to reductions in the use of rescue medication and the number of asthma exacerbations were also reported, there was limited or no evidence to suggest that there is a relationship between ACT score and general HRQoL, HRU, and healthcare costs.

Overall, these findings support the use of the ACT in a clinical setting, as a valid measure of disease control and associated patient outcomes, including ongoing symptomology and future risk. They also support the clinical use of the ACT to guide the appropriate management of patients with asthma, including when and how to select between alternative treatments. Additionally, the available evidence provides a foundation for the use of the ACT as a primary or secondary endpoint in clinical trials, allowing investigators to gauge accurately the effectiveness of a treatment.

The overall strength of this review is that it collates the published relationships between ACT score and a broad range of clinical outcomes into a coherent whole. By aiding our understanding of how ACT score is reflective of the different aspects of patients’ asthma experience, this review provides support for its use as a viable measure for other outcomes.

Limitations include the targeted nature of the literature search, which may not have encompassed the full body of literature on the correlation being investigated, and the presence of large differences in scientific rigor and reporting standards between the included articles. Additionally, the statistical power may have been inadequate in some of the evaluated studies, either by being insufficiently powered to evaluate the relationships between ACT score and the outcomes of interest, or overpowered to the extent such as a weak relationship became highly statistically significant.

With respect to future work, the exploratory setup of this research provides a characterization of the topics on which scientific data regarding the ACT are present or absent. As such, the current report provides a starting point to explore and corroborate these findings in future research initiatives on the value of ACT scores in real-world clinical settings. More studies evaluating relationships between ACT score and general HRQoL, healthcare costs, and resource use are also needed, as well as additional research into relationships in populations with differing levels of asthma severity.

## Conclusion

Despite some limitations inherent to the nature of a targeted literature review, this report provides an informative qualitative assessment of the available literature on the relationships between ACT score and a broad range of outcomes of interest, supporting the use of the ACT in clinical practice and trial settings.

## Data Availability

All data used in this review were taken from publicly available articles, and can be found at the relevant journal websites.

## Abbreviations

ACQ: Asthma Control Questionnaire
ACT: Asthma Control Test
ANOVA: Analysis of variance
AQLQ: Asthma Quality of Life Questionnaire
ATS: American Thoracic Society
CHEST: Annual meeting of the American College of Chest Physicians
EQ-5D: EuroQol five dimensions questionnaire
ERS: European Respiratory Society
FEV_1_: Forced expiratory volume in 1 s
FVC: Forced vital capacity
HRQoL: Health-related quality of life
HRU: Healthcare resource use
IMPALA: Impact on Work Productivity Index
MOS: Medical Outcomes Study
PSQI: Pittsburgh Sleep Quality Index
QoL: Quality of life
SABA: Short-acting beta agonist
SF-12: Short Form (12-item) Health Survey
WPAI: Work Productivity and Activity Impairment questionnaire
